# CD47: A Cell Surface Glycoprotein Which Regulates Multiple Functions of Hematopoietic Cells in Health and Disease

**DOI:** 10.1155/2013/614619

**Published:** 2013-01-21

**Authors:** Per-Arne Oldenborg

**Affiliations:** Section for Histology and Cell Biology, Department of Integrative Medical Biology, Umeå University, 901 87 Umeå, Sweden

## Abstract

Interactions between cells and their surroundings are important for proper function and homeostasis in a multicellular organism. These interactions can either be established between the cells and molecules in their extracellular milieu, but also involve interactions between cells. In all these situations, proteins in the plasma membranes are critically involved to relay information obtained from the exterior of the cell. The cell surface glycoprotein CD47 (integrin-associated protein (IAP)) was first identified as an important regulator of integrin function, but later also was shown to function in ways that do not necessarily involve integrins. Ligation of CD47 can induce intracellular signaling resulting in cell activation or cell death depending on the exact context. By binding to another cell surface glycoprotein, signal regulatory protein alpha (SIRP**α**), CD47 can regulate the function of cells in the monocyte/macrophage lineage. In this spotlight paper, several functions of CD47 will be reviewed, although some functions may be more briefly mentioned. Focus will be on the ways CD47 regulates hematopoietic cells and functions such as CD47 signaling, induction of apoptosis, and regulation of phagocytosis or cell-cell fusion.

## 1. Structure and Expression of CD47 in the Plasma Membrane

CD47 (originally named integrin-associated protein (IAP)) is a cell surface protein of the immunoglobulin (Ig) superfamily, which is heavily glycosylated and expressed by virtually all cells in the body [1]. CD47 was first recognized as a 50 kDa protein associated and copurified with the *α*_v_ *β*_3_ integrin in placenta and neutrophil granulocytes and later shown to have the capacity to regulate integrin function and the responsiveness of leukocytes to RGD-containing extracellular matrix proteins [1–4]. Soon after this integrin-associated protein was cloned, it was shown to be identical to the erythrocyte cell surface antigen CD47 [5]. The fact that CD47 is also expressed by cells like erythrocytes, that do not express integrins, indicates that it can be more appropriate to refer to this protein as CD47 than using its original name integrin-associated protein (IAP). The protein is fairly well conserved between species and has about 60–70% similarity in the amino acid sequence when comparing human CD47 with that of mouse, rat, and bovine CD47. CD47 has also been shown to be identical to the OA-3/OVTL3 antigen highly expressed on most ovarian carcinomas [6, 7]. It also shows homology to a protein family of variola and vaccinia viruses [1, 5, 6], the significance of which is still unclear. CD47 consists of an extracellular IgV domain, a five times transmembrane-spanning domain, and a short alternatively spliced cytoplasmic tail. In both humans and mice, the cytoplasmic tail can be found as four different splice isoforms ranging from 4 to 36 amino acids, showing different tissue expression patterns. The 16 amino acid form 2, which is by far the predominant isoform, is expressed in all cells of hematopoietic origin, as well as in endothelial and epithelial cells [8]. In contrast, the 36 amino acid form 4 is expressed primarily in neurons, intestine, and testis [8]. Expression of the 4 amino acid form 1 is found in epithelial and endothelial cells, while the expression pattern of the 23 amino acid form 3 resembles that of form 4 [8]. Despite a study showing that CD47 form 3 and 4 could be associated with memory retention in rats, and that form 2 is predominating in astrocytes [9], little is known as to what these splice variants mean in terms of possible difference in the functionality of the protein. A new twist in the understanding of CD47 comes from a recent finding that CD47 can be expressed as a proteoglycan with a molecular weight of >250 kDa, having both heparin and chondroitin sulfate glycosaminoglycan (GAG) chains [10]. This form of CD47 was found to be expressed in both the human Jurkat T cell line and in murine primary T cells, as well as in human umbilical vein endothelial cells (HUVECs), murine lung endothelial cells, and in human smooth muscle cells [10]. Functionally, the GAG chains at CD47 Ser^64^ were found to be crucial to inhibit T cell receptor signaling following the ligation of CD47 by its ligand thrombospondin-1 [10].

## 2. Intracellular Signaling following CD47 Interactions with Integrins, Thrombospondin (TSP), or Signal Regulatory Proteins (SIRPs)

### 2.1. Interaction with Integrins

**Figure 1:**
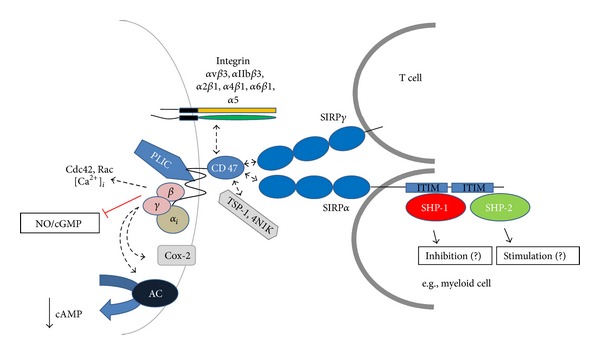
Interactions of CD47 in *cis* and *trans*. CD47 interacts in *cis* with integrins, in *trans* with SIRPs, and can also bind the soluble protein TSP-1. The figure summarizes intracellular signaling events associated with CD47 upon binding to its interaction partners.

Early studies of CD47 were based on the use of monoclonal antibodies (mAbs) raised against the CD47 protein purified from placenta [2, 11], showing a role of CD47 in mediating an enhanced IgG-mediated phagocytosis response in the presence of RGD-containing ligands, such as fibronectin, fibrinogen, vitronectin, or collagen type IV [2, 11]. The same mAbs were also found to block neutrophil transendothelial migration stimulated by interleukin 8 (IL-8) or the bacterial peptide N-formyl-methionyl-leucyl-phenylalanine (f-Met-Leu-Phe) and to inhibit neutrophil migration across tumor-necrosis-factor-*α*- (TNF*α*-) stimulated endothelial cells, where CD47 on both the neutrophils and the endothelial cells was found to be important [12]. Generation of other anti-CD47mAbs, raised against epithelial membrane preparations, showed that CD47 is present at the basolateral membrane of epithelial cell monolayers, that mAbs blocking CD47 on either neutrophils or the epithelial cells delay neutrophil *trans*-epithelial migration [13], and that efficient neutrophil chemotaxis correlates with an increased neutrophil cell surface expression of CD47 [14]. The basement membrane protein entactin, which contains an RGD sequence, was also found to stimulate neutrophil adhesion and chemotaxis in a CD47-dependent manner *in vitro* [15]. Generation of CD47-deficient mice further proved the importance of this protein in regulating neutrophil inflammatory responses, by showing an increased sensitivity to bacterial infection due to a delayed neutrophil accumulation in bacterial peritonitis [4]. CD47-deficient neutrophils also show a strongly impaired RGD-stimulated neutrophil adhesion, phagocytosis, and respiratory burst [4]. For *α*_v_ *β*_3_ integrin-mediated cellular responses to the extracellular matrix protein vitronectin, CD47 was found to be required for *α*_v_ *β*_3_-mediated binding to vitronectin-coated beads, but not *α*_v_ *β*_3_-mediated adhesion to vitronectin-coated surfaces [3]. In addition to its original association with *α*_v_ *β*_3_ integrins, CD47 has also been shown to interact with and regulate the integrins *α*_2_ *β*_1_ and *α*II_b_ *β*_3_ on platelets [16, 17], the *α*_2_ *β*_1_ integrin on smooth muscle cells [18], the *α*_4_ *β*_1_ integrin on sickle red blood cells and B lymphocytes [19, 20], the *α*_6_ *β*_1_ integrin in microglia [21], and the *α*_5_ integrin in chondrocytes [22] (Figure 1).

### 2.2. Interaction with Thrombospondin

Thrombospondin-1 (TSP-1) is the prototypic member of the thrombospondin family of extracellular matrix glycoproteins, which are implicated in regulating cell motility, proliferation, and differentiation [23]. The extracellular IgV domain of CD47 was found to be a receptor for the C-terminal cell-binding domain (CBD) of TSP-1, since the expression of CD47 in otherwise CD47-deficient cells promotes adhesion to TSP-1 or its CBD, and a functional blocking mAb against CD47 can block endothelial cell chemotaxis against TSP-1 or the CD47 binding CBD-peptide 4N1K [24]. It was later shown that TSP-1, its CBD, or the 4N1K peptide stimulates *α*_v_ *β*_3_ integrin-mediated cell spreading on vitronectin in a CD47-dependent manner [24]. In platelets, TSP-1, the TSP-1 CBD, or 4N1K activates the platelet *α*II_b_ *β*_3_ integrin and induces platelet spreading on fibrinogen, platelet aggregation, and increased focal adhesion kinase (FAK) tyrosine phosphorylation, which are all dependent on interaction between CD47 and integrin *α*II_b_ *β*_3_ [16]. Furthermore, CD47 was found to mediate a synergistic effect of soluble type I collagen and TSP-1 or 4N1K, which enhance *α*_2_ *β*_1_ integrin-mediated platelet activation or vascular smooth muscle cell chemotaxis [17, 18]. Early experiments also suggested that CD47 regulates TSP-1-induced cell spreading or platelet activation by affecting signal transduction in a pertussis toxin-sensitive way via a heterotrimeric Gi protein [16, 24]. CD47 was later shown to functionally associate with heterotrimeric Gi, to suppress cAMP levels, and mediate the inhibition of ERK in platelets and smooth muscle cells [25]. More recently, CD47 ligated by TSP-1 was found to inhibit nitric oxide (NO) signaling in vascular cells [26] and to oppose NO/cGMP-mediated inhibition of integrin activation to facilitate platelet aggregation [27].

In addition to signaling through Gi proteins, CD47 has been shown to signal via the *β*_3_ integrin cytoplasmic tail [28]. Although it is not entirely clear how much these two signaling pathways overlap, accumulation of CD47/*β*_3_-integrin-complexes in cholesterol-rich lipid rafts, which appears to depend on both the CD47 IgV domain, the multiple transmembrane-spanning domain, and a long range disulfide bond between Cys^33^ in the IgV domain and Cys^263^ in the transmembrane domain [29], engage in Gi signaling [30]. CD47 has also been shown to be involved in the regulation of intracellular Ca^2+^ ([Ca^2+^]_i_), exemplified by its regulation of an integrin-dependent increase in [Ca^2+^]_i_ in endothelial cells [31] and tumor cells [32], and that CD47 synergizes with T cell receptor-stimulated elevations of [Ca^2+^]_i_ in T lymphocytes [33, 34]. The cytoplasmic tails of the form 2 and form 4 splice variants of CD47 were found to bind to the cytosolic ubiquitin-related proteins PLIC-1 and PLIC-2 (PLIC, proteins linking IAP to cytoskeleton) [35]. In B lymphocytes, CD47 was suggested to synergistically promote cell migration trough Cdc42 [20]. A role of Cdc42 and Rac has also been suggested in CD47-dependent regulation of neuronal development and neurite formation [36, 37]. Adhesion of intestinal epithelial cells to collagen I induces the association of CD47 with *α*_2_ integrins, and CD47 is necessary for collagen I-induced cyclooxygenase-2 (Cox-2) expression and epithelial cell migration, which is mediated by G*α*_i3_ [38] (Figure 1).

### 2.3. Interaction with SIRP Proteins

SIRP proteins belong to the Ig family of cell surface glycoproteins, where the first member identified was SIRP*α* (also known as SHPS-1, CD172a, BIT, MFR, or P84) [39–44]. SIRP*α* is highly expressed in myeloid cells and neurons, but also in endothelial cells and fibroblasts, and has three extracellular Ig-like domains, one distal IgV-like domain, and two membrane proximal IgC-like domains [41, 42]. In addition, an alternatively spliced form having only one IgV domain has also been reported [45]. In its intracellular tail, SIRP*α* has two immunoreceptor tyrosine-based inhibitory motifs (ITIMs), which when tyrosine phosphorylated can bind the Src homology 2 (SH2) domain-containing protein-tyrosine phosphatases SHP-1 and SHP-2 [42]. Additional cytoplasmic binding partners for SIRP*α* are the adaptor molecules Src kinase-associated protein of 55 kDa homolog/SKAP2 (SKAP55hom/R), Fyn-binding protein/SLP-76-associated phosphoprotein of 130 kDa (FYB/SLAP-130), and the tyrosine kinase PYK2 [46]. SIRP*α* is also a substrate for the kinase activity of the insulin, EGF, and bPDGF receptors, and the overexpression of SIRP*α* in fibroblasts decreases proliferation and other downstream events in response to insulin, EGF, and bPDGF [42]. Since SIRP*α* is also constitutively associated with the M-CSF receptor c-fms, SIRP*α* overexpression partially reverses the v-fms phenotype [42]. Two other family members have also been identified, SIRP*β* (also known as CD172b) [42, 47] and SIRP*γ* (also known as CD172g or SIRP*β*2) [48], whose extracellular Ig-like domains are similar to that of SIRP*α*. However, the cytoplasmatic regions of SIRP*β* and SIRP*γ* are different from that of SIRP*α*. SIRP*β* has a very short cytoplasmatic tail with no signaling motifs. Instead, the transmembrane region contains a positively charged lysine residue, which can bind the immunoreceptor-tyrosine-based-activating-motif- (ITAM-) carrying adaptor protein DNAX activation protein 12 (DAP12/KARAP) [49, 50]. SIRP*γ* has no recognizable signaling motif or capability to interact with cytoplasmic signaling molecules and is therefore unlikely to generate intracellular signals [51]. CD47 has been shown to be a ligand for SIRP*α* [52, 53] and SIRP*γ* [54, 55], but does not bind SIRP*β* [47]. The CD47/SIRP*α* interaction regulates not only a multitude of intercellular interactions in many body systems, such as the immune system where it regulates lymphocyte homeostasis [56, 57], dendritic cell (DC) maturation and activation [58], proper localization of certain DC subsets in secondary lymphoid organs [59–61], and cellular transmigration [62, 63], but also regulates cells of the nervous system (reviewed in [64, 65]). An interaction between these two proteins also plays an important role in bone remodeling [66, 67]. Cellular responses regulated by the CD47/SIRP*α* interaction are many times dependent on a bidirectional signaling through both receptors [51, 64, 65] (Figure 1). The finding that CD47 on host cells can function as a “marker of self” and regulate phagocytosis by binding to SIRP*α* [68] will be further described in a subsequent section. The interaction between CD47 and SIRP*α* has proven to be very specific species, as shown by the relatively weak binding of CD47 from mouse, rat, or cow to human SIRP*α* [69, 70]. In addition, the glycosylation of CD47 or SIRP*α* does not seem to be necessary for their interaction [70], but the level of N-glycosylation of SIRP*α* has, however, an impact on the interaction such that over glycosylation reduces the binding of CD47 [71]. The long range disulfide bond between Cys^33^ in the CD47 IgV domain and Cys^263^ in the transmembrane domain is also important to establish an orientation of the CD47 IgV domain that enhances its binding to SIRP*α* [29].

## 3. CD47-Induced Apoptosis

**Figure 2:**
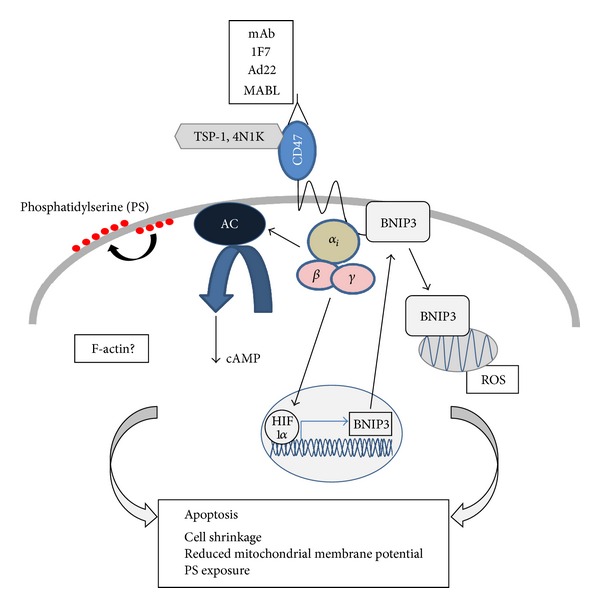
Mechanisms involved in mediating CD47-induced cell death. Ligation of CD47 by mAbs or TSP 1 induces apoptosis, which involves the activation of heterotrimeric Gi proteins, reduction in cAMP, and exposure of phosphatidylserine in the outer leaflet of the plasma membrane. It also induces the expression of the proapoptotic Bcl-2 family member BNIP3, its association with CD47, and subsequent translocation of BNIP3 to mitochondria. CD47-induced apoptosis may also involve ROS and F-actin.

Ligation of CD47 by anti-CD47 mAbs was found to induce apoptosis in a number of different cell types. This phenomenon was first described in Jurkat T cells, in anti-CD3 activated but not in resting primary T cells, and in B-cell chronic lymphocytic leukemia (B-CLL) cells [72, 73]. CD47-induced apoptosis can be induced by several different mAbs; however, while some of these (e.g., Ad22, 1F7, or MABL) show potent apoptosis induction in suspension [73–76], others (e.g., B6H12 and 2D3) need to be immobilized to a surface to promote cell death [72, 77]. Of the two SIRP-family members known to bind the CD47 IgV domain (SIRP*α* and SIRP*γ*), SIRP*α* as a soluble Fc-fusion protein does not induce CD47-dependent apoptosis [78], while SIRP*α* or SIRP*γ* bound onto the surface of beads induces apoptosis through CD47 in Jurkat T cells and the myelomonocytic cell line U937 [54]. In addition, TSP-1 or the CD47-binding TSP-1 CBD-peptide 4N1K also induces CD47-dependent apoptosis [74, 75, 78, 79]. Indeed, mice deficient in CD47 or TSP-1 sustain oxazolone-induced inflammation significantly longer than wild-type mice due to a deficiency in T cell apoptosis [79]. This form of cell death was initially described to be characterized by cell shrinkage, reduction in mitochondrial transmembrane potential, and exposure of phosphatidylserine (PS) on the cell surface, but to be independent of Fas (CD95) or TNF receptor signaling. Neither does CD47-induced cell death include DNA fragmentation nor other nuclear features associated with “classical” apoptosis, and it is independent of cysteinyl aspartate protease (caspase) activation [72, 73] (Figure 2). Furthermore, inhibitors of actin polymerization or mitochondrial electron transfer prevent CD47-induced PS exposure [77]. In support of a role for the actin cytoskeleton, peripheral blood mononuclear cells (PBMCs) from Wiskott-Aldrich syndrome (WAS) patients, where mutations in the WAS protein (WASP) results in defective Cdc42-induced regulation of the actin cytoskeleton [80], are resistant to CD47-induced apoptosis [77]. Although the mitochondrial transmembrane potential is affected in CD47-induced apoptosis, it does not involve the mitochondrial release of cytochrome c or apoptosis-inducing factor (AIF), but does involve the production of reactive oxygen species (ROS) [81]. In Jurkat T cells, it was shown that the inhibition of Gi*α* signaling with pertussis toxin can counteract CD47-induced apoptosis, that ligation of CD47 reduces intracellular cAMP levels, and that cAMP elevating agents prevents apoptosis by CD47 ligands [74]. This signaling pathway, which likely also involves reduced signaling through protein kinase A (PKA), is not only described in T cells, but also in several breast cancer cell lines [75]. In the latter cell type, it was shown that epidermal growth factor can inhibit the CD47 death pathway via protein kinase C (PKC) [75]. A yeast two-hybrid screen, where CD47 was used as bait, identified the pro-apoptotic Bcl-2 family member Bcl-2-homology-3- (BH3-) only protein 19 kDa interacting protein-3 (BNIP3) [78]. In T cells, BNIP3 was found to physically associate with CD47, which prevents its degradation in proteasomes and sensitizes T cells to CD47-induced apoptosis [78, 79]. Ligation of CD47 induces the translocation of BNIP3 to mitochondria, and attenuation of BNIP3 activity inhibits CD47-induced apoptosis [78], which together suggests that BNIP3 is crucial as a mediator of this cell death pathway. Moreover, BNIP3 gene expression was found to be increased and regulated by hypoxia-inducible factor-1*α* (HIF-1*α*) following the ligation of CD47 by single-chain fragments of an anti-CD47 mAb which kills B-CLL cell lines both *in vitro* and *in vivo*, where the knockdown of HIF-1*α* represses CD47-induced cell death [82] (Figure 2). The finding that a Jurkat T cell clone lacking CD47 is resistant to Fas- (CD95-) induced apoptosis, but that expression of CD47 restores the sensitivity to Fas-ligation, suggested that CD47 can augment Fas-induced apoptosis via a mechanism that requires neither CD47 signaling nor its association with lipid rafts [83]. In fact, the lack of CD47 impairs important proapoptotic events downstream of Fas, such as loss of mitochondrial membrane potential, cytochrome c release, caspase activation, poly(ADP-ribose) polymerase (PARP) cleavage, and DNA fragmentation [83]. This function of CD47 is likely also important in primary cells, since T cells from CD47-deficient mice are protected from Fas-induced apoptosis [83].

In hematopoietic cells, CD47-induced apoptosis has been described in hematopoietic tumor cells [54, 72–74, 76–78, 81, 82, 84, 85] and in activated primary T or B cells [73, 74, 77, 79]. However, whether apoptosis can be induced through CD47 in nonactivated leukocytes is still somewhat unclear. The only situation where nonactivated T or B cells have been found to undergo CD47-induced apoptosis is when immobilized anti-CD47 mAb has been used [77], but not when using soluble CD47 ligands or mAbs known to induce apoptosis in activated cells or tumor cells [73, 74, 79]. Surprisingly, although CD34^+^ hematopoietic progenitors express CD47, they are resistant to CD47-induced apoptosis by either immobilized or soluble mAb [76, 77]. In addition, immature human monocyte-derived dendritic cells (iDCs) were described as resistant to CD47-induced apoptosis, following incubation with an immobilized CD47 mAb for 18 hours [77]. However, another study showed that freshly isolated human monocytes or human monocyte-derived iDC undergoes a rapid (within 60 min) cell death in response to the CD47-ligand 4N1K [86]. This cell death, which was described to occur in a subset of cells and where monocytes or iDC not affected by 4N1K remain viable in culture, is associated with cellular features previously described for CD47-induced apoptosis, such as not only PS exposure, increased plasma membrane permeability, reduced mitochondrial membrane potential, caspase independence, but also included DNA fragmentation [86]. Thus, these findings suggest that although a subset of iDC may undergo CD47-induced cell death at an early time point, this may not be detectable at later time points. However, it raises the question if specific subsets of monocytes or iDCs are sensitive to this form of rapid cell death whereas others are resistant and maintain their viability in culture. In addition to hematopoietic cells, overexpression of CD47 can induces cell death of cultured cerebral cortical neurons, which is enhanced by the coexpression of SIRP*α* and prevented by brain-derived neurotrophic factor (BDNF) when CD47 and SIRP*α* are coexpressed [87]. Apoptosis in neurons overexpressing CD47, however, is dependent on caspases and apoptotic cells have condensed apoptotic nuclei with fragmented DNA [87]. It has also been shown that endothelial cells incubated under static conditions in the absence of flow increase their expression of TSP-1, which uses the CD47/*α*_v_ *β*_3_ integrin as a receptor to trigger endothelial cell apoptosis [88]. This mechanism also appears to be involved in endothelial cell apoptosis during proatherogenic turbulent flow conditions [89] and in mechanosensitive induction of apoptosis in fibroblasts [90]. TSP-1-mediated apoptosis, mapped to the type-3 repeat/c-terminal domain of TSP-1, in promyelocytic leukemia cells (NB4-LR1) has also been suggested to depend on the engagement of both CD47 and the *α*_v_ *β*_3_ integrin [91]. In contrast to the proapoptotic effects of CD47 described above, it was reported that the TSP-1-derived peptide 4N1 could abolish C_2_-ceramide-induced apoptosis in primary porcine thyroid cells by preventing reduction in intracellular cAMP levels, an effect blocked by the functional blocking antihuman CD47 mAb B6H12 [92]. A similar effect of 4N1 peptide was also found to inhibit the cytotoxic effects of the anticancer drugs camptothecin and doxorubicin in thyroid carcinoma cells [93]. Although it is unclear how these effects of CD47-ligation can be explained in relation to the proapoptotic effects of this molecule, it is interesting to note that tumor cell TSP-1 overexpression has been linked to disease recurrence and decreased survival [94–96], and it was suggested that this pathway could be one explanation behind drug resistance in thyroid cancers [93].

## 4. Expression of CD47 in Erythrocytes

### 4.1. Interaction of CD47 with Erythrocyte Membrane Protein Complexes

**Figure 3:**
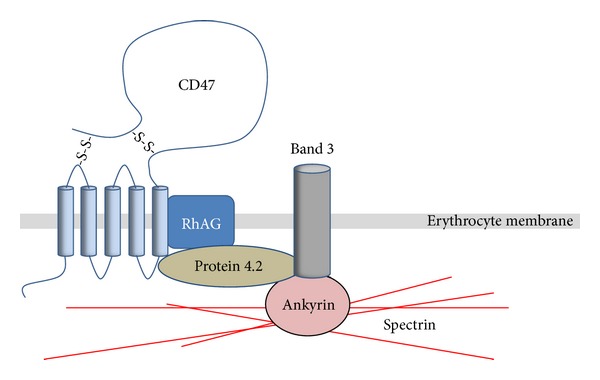
Association of CD47 with the Rh and band 3 complexes in the erythrocyte membrane. CD47 interacts with the Rh/RhAG complex and also associates with protein 4.2, which links CD47 to the band 3/ankyrin complex and the spectrin cytoskeleton. It is important to note that not all CD47 appears to be associated with this multiprotein complex in erythrocyte membranes.

Mature erythrocytes express high levels of CD47, but do not express integrins, which early indicated that other important functions of CD47 could be expected in these cells. The fact that individuals with the Rh_null_ phenotype, which do not express any of the proteins of the Rh protein complex, only express about 25% of normal levels of CD47 suggested a close relation between CD47 and erythrocyte Rh proteins [5, 97]. In the erythrocyte cell membrane, Rh polypeptides associate in a complex with many other proteins (e.g., Rh associated glycoprotein (RhAG), glycophorin B, LW, and CD47) [98]. Another erythrocyte membrane protein complex is formed by the band 3 anion exchanger and several other proteins (e.g., glycophorin A, protein 4.2, and ankyrin). The latter multiprotein complex mediates the anchorage of the erythrocyte membrane to the spectrin cytoskeleton [99, 100]. In addition, it has been suggested that the Rh complex and the band 3 complex may in fact be associated in the erythrocyte membrane [101]. Mutations in band 3, or complete band 3 deficiency, in human erythrocytes results in reduced expression of Rh polypeptides and RhAG [102] and results in a virtual lack of CD47 [101]. Moreover, human erythrocytes deficient in protein 4.2 also show a marked deficiency of CD47 as well as an altered glycosylation of RhAG [103]. When combining these finding, a hypothesis was put forward, which suggests that CD47 of the Rh complex may indeed form a link to the band 3 complex by binding to protein 4.2 [101] (Figure 3). Studies of protein mobility within the erythrocyte membrane have shown that CD47 is associated with the erythrocyte cytoskeleton as being a part of the Rh complex, but that CD47 is also present as a noncytoskeleton anchored pool which is more mobile in the erythrocyte membrane [104]. Thus, CD47 in erythrocytes may serve several different functions depending on its grade of mobility in the plasma membrane. It has been suggested that the freely mobile CD47 pool may be of importance to accumulate CD47 in specific membrane areas upon cell-cell contacts, for example, in order to efficiently interact with SIRP*α* on other cell types such as phagocytic cells [104]. Importantly, data from studies of murine erythrocytes have shown remarkable differences as compared with human erythrocytes, and the link between CD47, the Rh complex, and protein 4.2 in murine erythrocytes is not well understood. First, there are little or no Rh polypeptides in erythrocytes from band 3-deficient mice, while the expression of CD47 is only slightly reduced [101]. Second, erythrocytes from CD47-deficient mice were found to contain normal amounts of murine Rh and RhAG polypeptides [105]. Third, erythrocytes from protein 4.2-deficient mice have normal amounts of CD47 [106]. Taken together, the total picture suggests that CD47 seems to interact with Rh polypeptides and protein 4.2 in human erythrocytes, whereas the interaction between CD47, Rh polypeptides, and proteins of the band 3 complex in murine erythrocytes is still unclear.

### 4.2. CD47 in Stored or Aged Erythrocytes

A progressive decrease in the CD47 expression level has been observed on human erythrocytes stored under blood bank conditions at +4°C for more than 14 days [107–109] and in a mouse model of erythrocyte storage which tried to mimic human blood bank conditions [110]. However, the magnitude of reduction in CD47 expression was rather different, from a modest reduction of up to 6% at day 42 of storage [107], around 30% reduction on day 28 of storage [108], to a more than 50% reduction on day 14 of storage [109]. Furthermore, a recent study indicated that the CD47 levels of erythrocytes stored for 35 days were not different from that of fresh cells [111]. The discrepancy between these findings may have several explanations, such as the exact storage conditions or methods for the quantification of erythrocyte CD47 expression levels. In addition, the loss of CD47 may also be sensitive to leukocytes remaining in the erythrocyte concentrates. Indeed, a gradual loss of CD47 from erythrocytes during storage was observed in stored erythrocytes irrespective of whether the buffy coat was removed or not before storage, although buffy coat removal resulted in an increased expression level of CD47 at all time points tested, showing a significant correlation between the number of remaining leukocytes and erythrocyte CD47 levels [108]. A study of cryopreserved leukoreduced erythrocytes was unable to detect any effects on erythrocyte CD47 expression levels [112]. Microparticles released in the blood from blood cells or endothelial cells have recently gained interest due to their possible role in regulating a variety of normal or pathological biological functions [113]. Such microparticles are also released from erythrocytes during cryopreservation or storage at +4°C, and these microparticles do among other erythrocyte membrane proteins also carry CD47 [112, 114]. Although the presence of CD47 on these microparticles suggests a mechanism for the observed loss of CD47 from stored erythrocytes, soluble CD47 has also been detected in the supernatants of erythrocytes stored at +4°C [107]. In addition to a possible loss of CD47 on stored erythrocytes, a recent study also indicated that storage as well as experimental aging *in vitro* results in a conformational change of the CD47 protein [111]. This modification can be detected as a selectively increased binding of the anti-CD47 mAb 2D3, which recognizes an epitope different from the epitope involved in the binding of TSP-1 or SIRP*α* and recognized by mAb B6H12 [111]. Normal erythrocyte aging in circulation is rather complicated to study in humans. However, the use of mouse models has been informative to further understand if the level of CD47 is changing during erythrocyte aging. In these models, biotin is injected intravenously to label all blood cells at one or two specific time points [115, 116]. Analyses of circulating erythrocytes at later time points will then allow for discrimination between older biotin-positive and younger biotin-negative circulating erythrocytes [115, 116]. Using this approach, it has been found that CD47 is gradually lost from the surface of circulating murine erythrocytes, where the oldest erythrocytes may have up to 30% lower CD47 expression levels as compared with younger erythrocytes [115, 117].

### 4.3. CD47 in Sickle Cell or Gaucher Disease

Sickle cell disease, caused by a mutation in the hemoglobin *β* chain and a formation of insoluble intracellular aggregates of mutated hemoglobin, resulting in characteristic sickle shaped erythrocytes, is associated with severe vasoocclusive crisis [118, 119]. One important mechanism behind this pathology is the enhanced adhesion of sickle erythrocytes to vascular endothelium [118, 119]. CD47 was shown to play a role in the pathologic sequestration of sickle erythrocytes to vascular endothelium under shear stress by binding to TSP [120]. It has been found that the reticulocyte-enriched fractions of sickle erythrocytes are most efficiently binding to endothelial cells, that this function requires heterotrimeric G proteins and tyrosine kinase activity [121], and is mediated by *α*_4_ *β*_1_ integrins on the reticulocytes [19]. Interestingly, sickle erythrocytes also show an increased expression of the CD47 epitope recognized by mAb 2D3 [120]. In Gaucher's disease, a sphingolipidosis caused by glucocerebrosidase deficiency, macrophages accumulate glucosylceramide following the excess phagocytosis of erythrocytes, which converts splenic macrophages to pathogenic Gaucher cells [122]. Erythrocytes from patients with untreated Gaucher's disease have been found to have reduced levels of CD47, which can be reversed upon enzyme-replacement therapy [123]. It has been suggested that the anemia associated with the untreated disease can in part be explained by a combination of reduced CD47 levels together with other morphological erythrocyte abnormalities observed in this disease [123].

## 5. Self versus Non-Self Discrimination in the Innate Immune System and Regulation of Phagocytosis by the CD47/SIRP***α*** Interaction

### 5.1. Self versus Non-Self Discrimination

The distinction between self and non-self is central to the maintenance of integrity in a multicellular organism and allows for a powerful and successful elimination of potentially dangerous pathogens, while carefully preserving healthy host cells and tissues. This distinction has been well studied in the adaptive immune response, which is specialized in the recognition of foreign peptides by virtue of small modifications to major histocompatibility complex (MHC) molecules made by these peptides [124]. For the innate immune system, recognition is thought to be based on a large extent on the recognition of specific microbial structures, pathogen-associated molecular patterns (PAMPs) [125]. The innate immune system of the host organism has through the evolution developed a group of receptors, pattern recognition receptors (PRRs), which serves to specifically recognize PAMPs [125]. In this way, the host may utilize a certain number of receptors, encoded in the genome, for the recognition of various evolutionary stable molecular pathogen-associated structures. While some of the PRRs (like the mannose receptor (MR)) recognize PAMPs directly [125], others (like complement receptors (CRs)) are specialized to detect products generated secondary to PAMP recognition [126]. However, a system based on specific recognition of “foreign” is clearly flawed as it prevents the recognition of anything that is also present on the organism's own cells. This can be very elegantly circumvented by a defense system that has very broad recognition, when it is combined with specific molecules that mark host cells and tissues as “self.” Thus, recognition of “self” will inhibit the activation of innate immune cells, whereas the recognition of “missing self” allows for an immune response to proceed. In other words, a system where the own cells express a unique marker of self, not present on foreign cells, would make the distinction between self and foreign very simple. In that way, it would not matter if the organism's own cells express substances or ligands that are similar to those found on foreign cells/particles. Innate immune cells such as macrophages would only look for the presence of the “self marker” on the recognized particle, where the presence of self would release the recognized particle but the absence of self would allow for activation and destruction. This would significantly simplify the recognition process, as there would only be a need for a few broad specific recognition receptors instead of a countless number of foreign-specific receptors. It would also fulfill the criteria for a defense system that effectively recognize and destroy foreign objects but at the same time reduces the risk of damaging the organism's own structures. Such a system for macrophage activation would be analogous to the well-established “missing-self hypothesis” for natural killer (NK) cell activation [127]. NK cells recognize target cells by a range of activating receptors which also recognize ligands on many normal cells. However, in the NK cell system expression of self-MHC class I will protect the recognized cell via the ligation of NK cell inhibitory receptors specific for “self-” MHC class I [128]. It is, however, important to note, that NK cell inhibitory receptors are now described, which seem to have ligands other than MHC class I [129]. Upon the binding of self-MHC class I, ligated NK cell inhibitory receptors will recruit and activate the phosphatases SHP-1/SHP-2 that mediate the inhibition of NK cell activation [129]. NK cells thus spare cells whenever they express “markers of normal self,” and eliminate them when these markers are absent or inadequately expressed. Other leukocytes also express molecules related to NK cell inhibitory receptors, further suggesting that similar mechanisms are operative also, for example, in macrophage activation [130]. Many of these inhibitory receptors recognize MHC class I [130], but the “marker of self” could in principle be any ubiquitously expressed surface molecule.

### 5.2. CD47 as a Marker of “Self”

**Figure 4:**
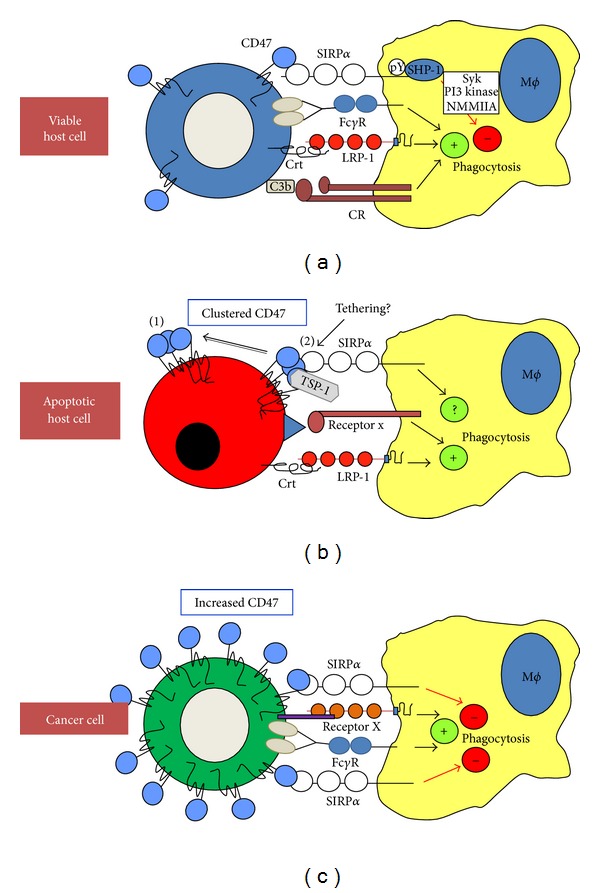
CD47 regulates phagocytosis of host cells by interacting with SIRP*α*. (a) CD47 on viable normal host cells can bind to SIRP*α* on a phagocytic cell (e.g., a macrophage (M*ϕ*)), which induces tyrosine phosphorylation of SIRP*α* ITIMS and recruitment of SHP-1. This can inhibit prophagocytic signaling through Fc*γ* receptors, complement receptors (CR), or LRP-1. Phagocytosis inhibition may involve signaling through Syk and PI3 kinase and inhibition of nonmuscle myosin type IIA (NMMIIA). (b) On apoptotic cells, CD47 becomes clustered in the plasma membrane and may be segregated away from domains containing ligands for prophagocytic receptors, here exemplified by calreticulin (crt) binding to LRP-1. However, most likely this principle involves also other prophagocytic receptors (receptor x). Clustered CD47 may also bind to SIRP*α* without inducing inhibition of phagocytosis, but may rather promote tethering of the apoptotic cell to the phagocyte. This function may also involve TSP-1 and so far uncharacterized mechanisms that can also promote phagocytosis. (c) Cancer cells may increase their expression of CD47 to strengthen the inhibitory signals through SIRP*α* and to more potently inhibit phagocytosis mediated by Fc*γ* receptors and other prophagocytic receptors (receptor x).

A new chapter in the understanding of CD47 and its functions started when CD47 was found to bind SIRP*α* [52, 53]. At the time, SIRP*α* was regarded as one of several immunoreceptors with cytoplasmic ITIM motifs, generally suggested to be involved in negative regulation of cellular functions, mediated by the recruitment of the tyrosine phosphatases SHP-1 and/or SHP-2 or the inositol phosphatase SHIP to the tyrosine phosphorylated ITIMs [131]. In addition, SIRP*α* was found to be highly expressed in primary macrophages and macrophage cell lines [45], as well as in other myeloid cells such as monocytes, granulocytes, and dendritic cells [53]. In macrophages, SIRP*α* was found to negatively regulate signaling through tyrosine kinase-dependent signaling pathways (e.g., Fc*ε*RI) [132]. Although both SHP-1 and SHP-2 can bind to the phosphorylated SIRP*α* ITIMs, only SHP-1 has been associated with the inhibitory function of SIRP*α* in macrophages, similar to that of the NK cell inhibitory receptors [128], whereas SHP-2 associated with SIRP*α* leads to a phosphatase-dependent enhancement of the signal in many situations [133]. When studying CD47-deficient mice, we were struck by the fact that CD47-deficient bone marrow cannot reconstitute and engraft in lethally irradiated syngeneic wild-type recipient mice, while it engrafts normally in CD47-deficient recipient mice [134]. Our work on trying to understand this controversy first involved experiments where different leukocyte populations were transferred from CD47-deficient mice into wild-type recipient mice. However, we later simplified the system by studying transfusion of erythrocytes instead of leukocytes. The reason for this is that erythrocytes in contrast to many leukocyte populations have a long half-life in circulation, they do not divide, do not express MHC class I, and they do not home to extravascular tissues or organs. These studies showed that fresh erythrocytes isolated from the blood of CD47-deficient mice, labeled with a fluorescent cell tracker dye and transfused into wild-type recipient mice, have markedly reduced survival, whereas their half-life is normal in CD47-deficient recipients [68]. The clearance rate of such freshly isolated CD47-deficient erythrocytes is remarkably fast with complete clearance within 24 hours [68]. To put this in perspective, the average lifespan of murine erythrocytes in circulation is somewhere between 45 and 60 days, depending on the mouse strain investigated [135, 136]. The rapid clearance of CD47-deficient erythrocytes from the circulation of wild-type recipient mice does not require complement, since CD47-deficient erythrocytes are also cleared from the circulation of complement factor 3- (C3-) deficient mice. Neither is there a requirement for lymphocytes or antibodies to enable the clearance of CD47-deficient erythrocytes from the circulation, since clearance is normal in Rag1-deficient mice, which lack mature T and B lymphocytes [68]. Rather, the transfused CD47-deficient erythrocytes that are eliminated from the circulation of wild-type mice are recognized and cleared by splenic red pulp macrophages, and the removal of these macrophages by splenectomy or by treatment with macrophage-toxic clodronate liposomes [137] abrogates the elimination of CD47-deficient erythrocytes [68]. Furthermore, macrophage SIRP*α* is tyrosine phosphorylated upon contact with CD47 on erythrocytes, and when SIRP*α* on isolated splenic macrophages is blocked, it increases the level of phagocytosis of wild-type erythrocytes to that seen with CD47-deficient erythrocytes, whereas phagocytosis of the CD47-deficient erythrocytes is unaffected by the antibody treatment [68]. In addition, studies in SIRP*α*-mutant mice, where the cytoplasmic signaling domain of the receptor is deleted, have shown a shorter half-life of normal CD47-expressing erythrocytes in these mice, which also present with mild anemia [138]. Such spontaneous anemia is, however, not seen in CD47-deficient mice, suggesting that CD47 could also be needed on the macrophages to facilitate the clearance of erythrocytes in the spleen. However, lack of CD47 on platelets also results in very rapid clearance when transfused into wild-type recipients, and both CD47-deficient mice and SIRP*α*-mutant mice have a mild spontaneous antibody-independent thrombocytopenia [139, 140]. Altogether, these findings indicated that all erythrocytes can be phagocytosed by splenic red pulp macrophages when SIRP*α* is blocked or CD47 is missing, and that these macrophages must have a receptor for erythrocytes. Indeed, we have identified LDL receptor-related protein (LRP-1), which by recognizing calreticulin on the surface of normal erythrocytes can mediate phagocytosis of untreated CD47-deficient erythrocytes [141, 142]. By showing that macrophages in the splenic red pulp can recognize normal circulating erythrocytes, but that these macrophages do not phagocytose erythrocytes as long as they display CD47 on their surface, these findings were the first to prove that macrophages are perfectly capable of recognizing “normal” host cells and rely on “self” recognition for proper function. In addition, it also demonstrated major similarities between mechanisms for “self” recognition in NK cells and macrophages [128]. Based on the findings that unopsonized erythrocytes lacking CD47 can be phagocytosed by splenic macrophages, and that the CD47/SIRP*α* interaction can potently inhibit the prophagocytic mechanism operating in that situation, we next hypothesized that the CD47/SIRP*α* interaction might as well be able to negatively regulate phagocytosis of opsonized erythrocytes, and that for the cells of the innate immune systems front line defense (i.e., macrophages and DC) there would be a balance between signals from activating receptors (e.g., Fc*γ*R or CR) and the inhibitory signal from SIRP*α* ligated by target cell CD47. Indeed, the CD47/SIRP*α* interaction can also regulate phagocytosis of IgG-opsonized or complement opsonized erythrocytes as well as other opsonized host cells, making CD47-deficient cells severely sensitive to being phagocytosed [139, 143–145]. In addition, CD47-deficient mice are severely sensitive to experimental autoimmune hemolytic anemia (AIHA) and experimental thrombocytopenia [139, 146]. On an autoimmune background prone to develop spontaneous AIHA, lack of CD47 results in a more rapid and lethal AIHA [146]. Using motheathen viable (me^v^/me^v^) mice, which only have about 20% of normal levels of SHP-1, but normal levels of SHP-2 [147–149], we could pinpoint the role of these phosphatases in mediating the phagocytosis inhibitory effect of the CD47/SIRP*α* interaction *in vivo*. When transfused into me^v^/me^v^ mice, IgG-opsonized wild-type erythrocytes are cleared with the same rapid kinetics as seen with equally opsonized CD47-deficient erythrocytes [145], showing that at that particular signaling strength through prophagocytic Fc*γ* receptors, the level of CD47 on normal erythrocytes was not enough to prevent phagocytosis if the SHP-1 level was reduced by about 80%. In contrast, the prophagocytic signaling mediating phagocytosis of unopsonized CD47-deficient erythrocytes (presumably mediated by LRP-1) is likely much weaker, since unopsonized wild-type erythrocytes are not cleared with the same rapid kinetics as CD47-deficient cells in me^v^/me^v^ mice [145]. However, while unopsonized CD47 heterozygous erythrocytes (expressing about 50% of the CD47 found on wild-type erythrocytes) show normal half-life when transfused into wild-type recipients, they were found to be cleared more rapidly when transfused into me^v^/me^v^ mice [145]. These findings thus proposed that a reduction of the CD47 level to 50% of normal, in combination with a strongly reduced level of SHP-1 in the macrophages, together weakened the inhibitory signaling through SIRP*α* such that clearance of unopsonized erythrocytes was allowed. Furthermore, the rate of phagocytosis of IgG-opsonized erythrocytes is distinctly regulated by the amount of CD47 present on the surface of the erythrocytes both *in vivo* and *in vitro* [150]. Thus, activation of phagocytosis in a macrophage in contact with a target erythrocyte (or any other host cell) can be viewed as a balance between signals from activating receptors (i.e., Fc*γ*R, CR, or LRP-1) and the inhibitory signal from SIRP*α* ligated by target cell CD47. In the macrophage, neither signal appears to be dominant, but rather the decision to phagocytose a target host cell is based on an integration of positive prophagocytic signals and inhibitory CD47/SIRP*α* signaling (Figure 4(a)). The same functional regulation also seems to be operating in DCs [134] and in microglia [151]. In hemophagocytic lymphohistiocytosis, hematopoietic stem cells are phagocytosed by bone marrow macrophages as a result of systemic inflammation [152]. In this disease, hematopoietic stem cells were found to express reduced levels of CD47 [153], which shows that pathological conditions may occur where a combination of inflammatory macrophage activation and reduced expression of CD47 results in a severe loss of critical cell types [153]. However, not all prophagocytic receptors seem to be regulated by the CD47/SIRP*α* interaction. Although Fc*γ* receptor-mediated phagocytosis of IgG-opsonized oxidatively damaged erythrocytes is strongly inhibited by the CD47/SIRP*α* interaction, scavenger receptor-mediated uptake of unopsonized oxidized erythrocytes turned out to be insensitive to this inhibitory mechanism [154]. The mechanism whereby recruitment of SHP-1 to SIRP*α* can inhibit phagocytosis has been suggested to involve the tyrosine kinase Syk and phosphoinositide 3 kinase (PI3 kinase) [144]. More recently, it was suggested that SHP-1 mediates dephosphorylation of nonmuscular myosin IIA at the phagocytic synapse between the phagocyte and a host cell, as a result of the interaction between macrophage SIRP*α* and CD47 on the host cell, which brings further insight into the mechanism behind phagocytosis inhibition by the CD47/SIRP*α* interaction [155].

Based on the seemingly important interaction between CD47 and SIRP*α* to prevent phagocytosis of host cells, and the fact that CD47-deficient cells are rapidly phagocytosed when transfused into wild-type mice, it is clearly a puzzle why CD47-deficient mice do not present with a more severe phenotype where the macrophages phagocytose a large fraction of the CD47-deficient cells in those mice [68]. However, this phenomenon is rather similar to what is described in *β*2 microglobulin deficient mice, which lack expression of MHC class I but where the NK cells still do not attack and destroy host cells and also show hyporeactivity to cells carrying ligands for activating NK cell receptors, cells which are efficiently killed by wild-type NK cells [156]. One explanation proposed for this function in NK cells is that inhibitory NK cell receptors need to interact with “self-” MHC class I in order for the NK cell to become “licenced” and able to become activated and kill target cells lacking or expressing reduced levels of “self-” MHC class I [156]. By investigating the ability to phagocytose CD47-deficient cells in mice where CD47 was expressed by hematopoietic cells, nonhematopoietic cells, or both, the phagocytic “tolerance” to cells lacking CD47 in CD47-deficient mice was recently proposed to function in a similar way [157]. It was found that macrophages developing in an environment where nonhematopoietic cells lack CD47 become “tolerant” to cells lacking CD47, which allowed CD47-defcient leukocytes to avoid clearance. Curiously, this did not involve erythrocytes, which were cleared by splenic macrophages also in the bone marrow chimeras where nonhematopoietic cells lacked CD47 and macrophages had become “tolerant” [157]. The latter phenomenon has so far not been explained, but may suggest that phagocytosis of erythrocytes is regulated differently from that of leukocytes. Although CD47-deficient macrophages express normal amounts of SIRP*α*, which can also become tyrosine phosphorylated upon contact with CD47 expressing cells (Oldenborg et al., unpublished observations and [158]), it is possible that the “tolerance” to CD47-deficient cells that develop in CD47-deficient mice has to do with tuning of intracellular signaling pathways. In favor of such a hypothesis is the observation that transfused IgG-opsonized wild-type erythrocytes are cleared from the circulation at a significantly slower rate in CD47-deficient mice, as compared with that seen in wild-type recipient mice (Oldenborg et al. unpublished observations). This is unlikely due to a different expression of Fc*γ* receptors in CD47-deficient mice, since CD47-deficient IgG-opsonized erythrocytes are cleared with the same kinetics in both wild-type and CD47-deficient recipient mice (Oldenborg et al. unpublished observations).

### 5.3. CD47 and Phagocytosis of Apoptotic Cells

Apoptosis is a physiological process of programmed cell death which is important for embryologic development, maintenance of homeostasis, and elimination of damaged cells. An important event related to this process is the rapid uptake of apoptotic cells or apoptotic bodies by phagocytic cells. The efficacy of this process in the body is shown by the fact that apoptotic cells are apparently removed with such extremely high efficiency that apoptotic cells are very hard to detect in tissues under normal physiological conditions. It has even been suggested that if apoptotic cells are in fact detected *in vivo*, this may indicate a possibility of defects in their clearance or the presence of a large overload of apoptotic cells [159–162]. The largest part of apoptotic cell phagocytosis is mediated not only by professional phagocytes like macrophages and DCs, but also to some extent by nonprofessional phagocytes such as fibroblasts; epithelial cells and stromal cells are equipped with this function [160]. One example of the latter phenomenon is the phagocytosis of apoptotic mammary epithelial cells by bystander epithelial cells during mammary gland involution [163, 164]. When CD47 was identified as a cell surface protein on host cells that can negatively regulate their phagocytosis through SIRP*α*, one important question was to understand if this signaling pathway is altered during apoptosis to facilitate uptake of apoptotic cells. One hypothesis would be that apoptotic cells downregulate the amount of CD47 on their surface, which together with an increased amount of prophagocytic ligands exposed during apoptosis would result in phagocytosis of the dead cells. Indeed we found a reduced expression of CD47 on apoptotic fibroblasts and neutrophils [141], but curiously not on apoptotic Jurkat T cells [141] or apoptotic murine thymocytes [165]. This issue became even more confusing when apoptotic murine T cells lacking CD47, in contrast to the solid data showing that CD47 on host cells inhibits phagocytosis by macrophages, were found to be phagocytosed less efficiently by macrophages than equally apoptotic CD47 wild-type T cells [143, 165]. In apoptotic murine T cells, CD47 was rather found to be important in mediating tethering of the apoptotic cells to the phagocyte [143, 165]. In addition, CD47 is redistributed into patches on the apoptotic cell surface, areas of the plasma membrane which are different than those harbouring clusters of proteins that function as ligands for prophagocytic receptors [141, 143]. Thus, one possibility would be that the segregation of CD47 away from these phagocytosis promoting ligands could allow for tethering mediated by the CD47/SIRP*α* interaction, but that SIRP*α* would be too far away from the prophagocytic receptors to be involved in negative regulation (Figure 4(b)). Another very interesting hypothesis was recently suggested, which shed new light on this process. As described above in Section 4.2 experimentally oxidized erythrocytes, as well as erythrocytes stored in blood bank conditions, showed enhanced expression of the CD47 epitope recognized by mAb 2D3, suggesting a conformational change in the CD47 IgV domain [111]. Importantly, this is associated with an increased binding of TSP-1 to CD47, which together could generate a new binding site for SIRP*α* that induces a prophagocytic signal instead of the “normal” inhibitory CD47/SIRP*α* signal [111]. However, this very interesting hypothesis still has to also be investigated in the context of macrophage phagocytosis of apoptotic nucleated cells such as neutrophils or T cells. Thus, CD47 may operate in several different ways during cellular senescence in order to switch its normal phagocytosis-preventing role to rather facilitate and promote the phagocytosis of senescent cells. LRP-1 is a multifunctional scavenger receptor, shown to be involved in mediating uptake of apoptotic cells [141, 166, 167], and as already mentioned it can also bind to calreticulin on viable erythrocytes to induce phagocytosis if the inhibitory CD47/SIRP*α* interaction is not strong enough [141, 142] (Figure 4(b)). Glucocorticoids are powerful in treatment of inflammatory conditions, which can be attributed to their ability to downregulate production of and cellular responses to proinflammatory cytokines and their ability to inhibit the recruitment of inflammatory cells [168, 169]. However, another mechanism whereby glucocorticoids may also reduce inflammation is to stimulate phagocytosis of apoptotic cells [170, 171]. Interestingly, glucocorticoid treatment of macrophages results in increased macrophage expression of LRP-1 and increased phagocytosis of apoptotic cells or viable CD47-deficient erythrocytes [142].

### 5.4. CD47 and Regulation of Tumor Cell Phagocytosis

Since CD47 can inhibit phagocytosis of host cells, and the amount of CD47 on the cell surface is clearly important in determining the phagocytosis efficiency of macrophages, this also raised the question on whether cells such as tumor cells can also increase their CD47 expression levels in order to escape macrophage elimination. Indeed, early studies showed that ovarian carcinoma cell lines express increased levels of CD47 [6, 7], the functional significance of which was unclear at that time. More recently, an enhanced expression of CD47 has been reported for murine myeloid leukemias, as well as human normal and leukemic hematopoietic stem cells and many human solid tumors [172–174], and that increased CD47 expression is associated with reduced patient survival in AML and solid tumors [173, 174]. To challenge the hypothesis that CD47 on the leukemic cells protects from phagocytosis by macrophages and supports tumor cell growth, a xenogenic mouse model was used to study a particular human AML clone (MOLM-13) with very low endogenous CD47 expression. Expression of murine CD47 in MOLM-13 cells and transplantation of MOLM-13 clones expressing low or high levels of CD47 showed an enrichment and spreading of the CD47-high expressing clones to many bones of recipient mice, while the CD47-low clones showed minimal engraftment [172]. Depletion of macrophages in recipient mice allows for engraftment of CD47-low clones, and macrophage phagocytosis of AML cells both *in vitro* and *in vivo* is selective for tumor cells expressing low amounts of CD47 [172]. Importantly, by blocking SIRP*α* on macrophages, phagocytosis of CD47-high AML cells is increased to that seen with CD47-low clones [172]. Thus, these findings strongly suggest that an increased CD47 expression level on tumor cells could serve to avoid macrophage clearance and promote dissemination of tumor cells (Figure 4(c)). It was also recently shown that treatment serving to block the CD47/SIRP*α* interaction can result in elimination of AML stem cells in a xenogenic model [175]. Furthermore, inline with our original finding that the CD47/SIRP*α* pathway also potently inhibits Fc*γ* receptor-mediated phagocytosis of IgG-opsonized host cells [139, 145, 146, 150], it has been shown that antibody-mediated blocking of CD47/SIRP*α* signaling promotes phagocytosis of non-Hodgkin lymphoma cells treated with rituximab [176] and antibody-dependent cellular cytotoxicity (ADCC) against Her2/Neu-positive breast cancer cells treated with traztusumab [177]. Thus, blocking the CD47/SIRP*α* interaction may prove to be a powerful tool in the treatment of various tumors.

### 5.5. CD47 and Regulation of Xenografts

While organ transplantation is an important procedure to treat end-stage organ failure, it is hampered by a shortage of organ donors. To solve this problem, it has been suggested that pigs could serve as donors of organs, tissues, or cells to be transplanted into humans (i.e., xenotransplantation) [158, 178]. Both the innate and adaptive immune system participates in the rejection of xenogenic transplants [178], the exact details of which is outside the scope of the present paper. However, of particular interest here are the findings suggesting that even in the absence of a functional adaptive immune system and in the absence of NK cells (both of which react to cell surface determinants of xenogenic cells recognized as non-self), rejection of xenogenic cells and tissues is seen, which suggests that macrophages could play an important role in this process [158]. Indeed, in studies of transplantation of pancreatic islets [179, 180] or hematopoietic cells [181] from pigs to mice, macrophage depletion significantly enhances engraftment. The ability of macrophage SIRP*α* to inhibit phagocytosis by binding to CD47 on a target cell, and the fact that this interaction is rather species specific suggested that this interaction could play a role in macrophage-mediated clearance of xenogenic cells. Indeed, it was found that porcine CD47 cannot bind to murine SIRP*α* and induce tyrosine phosphorylation of the SIRP*α* ITIMs [182], which could explain the rapid clearance of porcine hematopoietic cells by macrophages both *in vitro* and *in vivo* [181, 182]. However, expression of murine CD47 in porcine cells markedly inhibits macrophage-mediated phagocytosis of these cells *in vitro* and prolongs their survival *in vivo* [182]. Although a human SIRP*α* fusion protein was found to bind to porcine CD47 [69], porcine CD47 does not activate human SIRP*α* [183]. As a result, porcine hematopoietic cells are rapidly phagocytosed by human macrophages, but expression of human CD47 in porcine cells results in attenuated phagocytosis by human macrophages [183]. These findings indicate that transgenic pigs expressing human CD47 could be an important possibility to further help reducing the rejection of porcine cells in xenotransplantation.

## 6. CD47 in Bone Homeostasis

The finding that CD47, by binding to SIRP*α*, could regulate rat alveolar macrophage fusion and formation of multinucleated giant cells *in vitro* [184] suggested a function also in regulating formation of bone-resorbing osteoclasts and regulation of bone homeostasis. Using functional blocking monoclonal antibodies against either CD47 or SIRP*α*, the formation of tartrate resistant acid phosphatase (TRAP)^+^ multinucleated osteoclasts is strongly inhibited in murine cell culture systems *in vitro* [66]. This finding can also be confirmed *in vivo*, since CD47-deficient mice have reduced numbers of osteoclasts in bone [66] and are protected from tumor metastasis and subsequent bone resorption [185]. There are a few hypotheses presented to explain the mechanism whereby the interaction between CD47 and SIRP*α* promotes osteoclastogenesis. One suggests that binding of CD47 results in SIRP*α* tyrosine phosphorylation and subsequent recruitment of SHP-1 to the phosphorylated ITIMs, which next would mediate dephosphorylation of nonmuscle cell myosin IIA to promote fusion and formation of osteoclasts [67]. This hypothesis is contrasted by a study of SIRP*α* mutant mice, expressing a truncated nonsignaling SIRP*α* cytoplasmic domain, where cultures of bone marrow cells generated the same number of osteoclasts of the same size, as found in wild-type bone marrow cultures [186]. The important observation made in the latter study was that osteoclasts generated in SIRP*α* mutant mice had an increased bone resorbing activity, suggesting that SIRP*α* negatively regulates this function in osteoclasts [186]. The contradictory findings presented regarding a possible role of signaling through either CD47 or SIRP*α* during fusion of preosteoclasts and formation of multinucleated osteoclasts must also be put in context of the originally presented hypothesis that the interaction between CD47 and SIRP*α* may mainly facilitate cell fusion by promoting cell-cell adhesion and bringing the plasma membranes of two cells close enough to facilitate cell fusion [187]. Here it has also been highlighted that CD47 may interact with the shorter SIRP*α* isoform having only one extracellular IgV domain, which would further reduce the distance between two opposing cellular membranes where cell fusion could take place [187].

## 7. Concluding Remarks and Future Perspectives

CD47 can regulate many important physiological cellular mechanisms by interacting with integrins, TSP-1, or SIRP*α*. As outlined above, much knowledge has been collected over the past two decades, but much is probably still to be discovered regarding the functions of this protein. There are several areas where one can assume that further progress may result in new novel ways to interfere with biological mechanisms in pathological conditions. One such example is the ability to induce apoptosis by ligating CD47. Although CD47 is expressed by virtually all cells in the body, in healthy as well as pathological cells, the data so far indicates that CD47-induced apoptosis may still be a way to kill, for example, tumor cells, since it appears that tumor cells and activated cells are much more sensitive to this cell death mechanism than nonactivated naïve cells or hematopoietic stem cells. The ability of CD47 to inhibit phagocytosis is clearly an important mechanism whereby innate cells as macrophages or dendritic cells can discriminate between self and non-self, and maintain tolerance to host cells. In the field of xenotransplantation, further development and research to create xenotransplants carrying human CD47 may prove important to obtain even better conditions where xenogenic cell, tissues, or organs can be tolerated when transplanted into humans. In the field of cancer research, where tumor cells may express higher levels of CD47 as a way of avoiding phagocytosis and clearance by phagocytic cells, and where CD47-blocking antibodies have been shown to promote clearance of and to reduce the dissemination of tumor cells, brings hope that careful development of reagents that can block the CD47/SIRP*α* interaction may indeed be useful to treat many forms of cancer without having too much of a negative side effect in terms of inducing clearance of host cells. Also in the field of bone research, one may suggest that manipulation of the interaction between CD47 and SIRP*α* may be a way to modulate bone resorption and to prevent osteoporosis. However, data showing that these two proteins may also be involved in regulating bone formation [67] indicate that manipulation of this signaling system in bone tissue may be more complicated.
